# Serum proteome profiling identified thrombospondin-1 and lactoferrin as biomarkers of relapsed multiple myeloma

**DOI:** 10.3389/fmed.2025.1640245

**Published:** 2025-09-08

**Authors:** Xiaoxiao Wu, Jianying Guo, Haiteng Deng, Wenming Chen

**Affiliations:** ^1^Department of Comprehensive Oncology, National Cancer Center/National Clinical Research Center for Cancer/Cancer Hospital, Chinese Academy of Medical Sciences and Peking Union Medical College, Beijing, China; ^2^MOE Key Laboratory of Bioinformatics, School of Life Sciences, Tsinghua University, Beijing, China; ^3^Department of Hematology, Beijing Chaoyang Hospital, Capital Medical University, Beijing, China

**Keywords:** THBS1, LTF, relapsed/refractory myeloma, proteomics, serum biomarker

## Abstract

**Introduction:**

Multiple myeloma (MM) remains an incurable hematologic malignancy characterized by inevitable relapse despite advances in novel therapeutics. Identifying reliable biomarkers for relapsed/refractory MM (RRMM) is crucial to improving clinical outcomes.

**Methods:**

We conducted a comprehensive proteomic analysis of bone marrow and peripheral serum samples from newly diagnosed MM (NDMM), RRMM, and MM remission patients, along with healthy controls. Using tandem mass tag (TMT)-labeled quantitative mass spectrometry, we quantified over 1,000 serum proteins. Identified candidate proteins were further validated via ELISA.

**Results:**

Thrombospondin-1 (THBS1) and lactoferrin (LTF) were significantly downregulated in the bone marrow serum of RRMM patients. ELISA validation confirmed markedly reduced levels of THBS1 and LTF in both bone marrow and peripheral serum of RRMM patients compared to NDMM, remission, and healthy control groups.

**Discussion:**

Our integrated proteomic and biochemical analyses suggest that the THBS1/LTF protein signature may serve as a predictive biomarker for MM relapse. This signature offers potential clinical utility in disease monitoring and therapeutic stratification.

## 1 Introduction

Multiple myeloma (MM) is a malignant proliferation of plasma cells characterized by excessive production of monoclonal immunoglobulins (M proteins). While novel therapeutic regimens have significantly improved complete remission (CR) rates, disease relapse remains inevitable due to either minimal residual disease (MRD) persisting after CR or the emergence of treatment-resistant clones.

Current diagnostic and monitoring strategies primarily rely on invasive bone marrow biopsies for morphological assessment, flow cytometry, and next-generation sequencing (NGS) of immunoglobulin gene rearrangements. Although sensitive, these approaches are impractical for frequent monitoring. While serum M-protein electrophoresis serves as a non-invasive monitoring tool, its clinical utility is limited by stable M-protein levels in many patients who subsequently develop relapsed/refractory MM (RRMM). Consequently, there is an urgent need for specific, non-invasive serum biomarkers to facilitate early detection of RRMM and guide therapeutic decision-making.

Mass spectrometry (MS)-based proteomics is a sensitive and high-throughput technique for detecting and identifying biomarkers. In a recent study, MS was used to detect the unique quality of a patient’s M-protein to monitor their disease over time (1), and serum sampling is less invasive than bone marrow biopsy. Therefore, serum-based MS is a desirable alternative for disease monitoring. It is urgently needed to identify differentially proteins between newly diagnosed MM (NDMM) and RRMM by proteomic studies, which may improve our understanding of crucial proteins associated with MM relapse. In this study, the proteomes of the bone marrow serum of patients with NDMM and RRMM were analyzed to identify key proteins that may have diagnostic value for RRMM. Additionally, we determined whether thrombospondin-1 (THBS1) and lactoferrin (LTF) were essential for MM recurrence.

THBS1, a 450-kDa extracellular matrix glycoprotein secreted by endothelial cells, fibroblasts, macrophages, monocytes, and some tumor cells (2), is associated with various cancer types.

The increase of serum THBS1 can inhibit the secretion of vascular endothelial growth factor (VEGF) (3). The expression level of THBS1 depends on the type of tumor. TGF-β1 induces THBS1 expression via Smad3, which contributes to the invasive behavior during glioblastoma expansion (4). THBS1, which is low expressed in prostate cancer, promotes neuroendocrine differentiation through CREB- EZH2-TSP1 pathway (5), and then promotes tumor progression.

LTF is an 80-kDa iron-binding glycoprotein (6) that protects against microbial pathogens in innate immunity. LTF is involved in many biological functions such as anti-inflammatory, anti-oxidative, and anti-tumor processes (6). Recent evidence indicated that LTF can influence the cell cycle process, regulate the phosphorylation of various kinases, and activate the VEGF receptor 2 (VEGFR2)-PI3K/AKT-ERK1 pathway (7), and the protein promotes mitochondrial apoptosis by upregulating the expression of Fas and Bid. All of these processes affect the growth and apoptosis of cancer cells. This led to our hypothesis that THBS1 and LTF play important roles in adjusting the progression of many tumors, making it vital to explore the influence of THBS1 and LTF expression on RRMM progression.

Through comprehensive proteomic profiling, we analyzed the expression patterns of thrombospondin-1 (THBS1) and lactoferrin (LTF) in bone marrow serum samples obtained from newly diagnosed (NDMM) and relapsed/refractory multiple myeloma (RRMM) patients. Our quantitative proteomic analysis demonstrated significantly reduced expression levels of both THBS1 and LTF in RRMM patients compared to their NDMM counterparts.

To further validate these findings, we systematically examined THBS1 and LTF expression across four distinct cohorts: NDMM patients, RRMM patients, MM patients in remission, and healthy controls. Remarkably, the RRMM group exhibited the lowest levels of both biomarkers among all study groups, suggesting a potential association between THBS1/LTF downregulation and disease progression to the relapsed/refractory state.

## 2 Materials and methods

### 2.1 Sample collection and processing

Bone marrow serum samples were obtained from patients with newly diagnosed multiple myeloma (NDMM; *n* = 9) and relapsed/refractory multiple myeloma (RRMM; *n* = 8). Peripheral blood serum samples were collected from additional cohorts including NDMM patients (*n* = 36), RRMM patients (*n* = 12), MM patients in remission (*n* = 36), and healthy controls (*n* = 36). All myeloma diagnoses were rigorously established according to the International Myeloma Working Group (IMWG) diagnostic criteria and verified against NCCN guidelines.

The study protocol received ethical approval from the Institutional Review Board of Beijing Chaoyang Hospital, Capital Medical University (Beijing, China). Following collection, all specimens were immediately centrifuged at 1,300 rpm for 10 min to separate serum components, then aliquoted and stored at −80°C until proteomic analysis to preserve sample integrity.

#### 2.1.1 Clinical characteristics

Detailed demographic and clinical parameters of all study participants, including disease stage, treatment history, and laboratory findings, are comprehensively documented in Supplementary Table 1 (bone marrow cohort) and Supplementary Table 2 (peripheral blood cohort). The patient selection process ensured strict adherence to both IMWG and NCCN diagnostic standards, maintaining consistency across all study groups.

### 2.2 Quantitative proteomic analysis

#### 2.2.1 Sample preparation and processing

High-abundance proteins were first depleted from clinical serum samples using a SepproIgY14 column (Becton, Dickinson and Company) following manufacturer’s protocol. Subsequently, 100 μg of protein lysates from each NDMM and RRMM sample were subjected to reduction with 5 mM dithiothreitol (DTT; Solarbio) and alkylation with 12.5 mM iodoacetamide (IAM; Solarbio). Proteins were then digested overnight at 37 °C with sequencing-grade modified trypsin (Promega) at a 1:50 enzyme-to-protein ratio. The resulting peptides were desalted using Sep-Pak C18 Vac cartridges (Waters) and dried by SpeedVac (Labconco) centrifugation.

#### 2.2.2 TMT labeling and fractionation

Dried peptides were reconstituted in 100 mM triethylammonium bicarbonate (TEAB; Sigma-Aldrich) and labeled with tandem mass tag (TMT) reagents (Thermo Scientific) according to the following scheme: NDMM samples were labeled with TMT6−126, −127, and −128 tags, while RRMM samples received TMT6−129, −130, and −131 tags. The labeling reaction was quenched after 1 h with 5% hydroxylamine (Thermo Fisher Scientific) for 15 min. All TMT-labeled peptides were pooled, desalted, and fractionated using a Thermo-Dionex Ultimate 3000 HPLC system equipped with a reverse-phase capillary column (75 μm × 150 mm, packed with 5-μm C18 resin, 300 Å pore size; Varian). Peptides were eluted over a 120-min gradient at 250 nL/min using mobile phase A (0.1% formic acid in water) and B (0.1% formic acid in acetonitrile).

#### 2.2.3 Mass spectrometry analysis

LC-MS/MS analysis was performed on a Q-Exactive mass spectrometer (Thermo Scientific) operated in data-dependent acquisition mode. Full-scan MS spectra (m/z 300–1800) were acquired in the Orbitrap at 70,000 resolution, followed by higher-energy collisional dissociation (HCD) of the top 20 most intense ions for MS/MS analysis.

#### 2.2.4 Data processing

Raw MS/MS data were processed using Proteome Discoverer software (v2.1) with the SEQUEST search engine against the UniProt human protein database. The mass spectrometry proteomics data have been deposited to the Proteome Xchange Consortium^1^ via the iProX partner repository with the dataset identifier PXD045583.

### 2.3 Enzyme-linked immunosorbent assay (ELISA).

Quantitative analysis of THBS1 and LTF protein levels was performed using commercially available ELISA kits (Abcam, Cambridge, UK; Catalog ab193716-1, ab200015-1) following the manufacturer’s standardized protocol. Briefly, serum samples were diluted appropriately and incubated in antibody-coated wells alongside provided standards. After thorough washing to remove unbound proteins, detection antibodies were applied followed by horseradish peroxidase (HRP)-conjugated secondary antibodies. The enzymatic reaction was developed using 3,3′,5,5′-tetramethylbenzidine (TMB) substrate, and the reaction was stopped at the optimal time point.

Absorbance measurements were obtained at 450 nm using a microplate reader (Thermo Fisher) within 5 min of reaction termination to ensure measurement accuracy. Protein concentrations were determined by interpolating optical density (OD) values against the standard curve generated from known concentrations of recombinant proteins. All samples were analyzed in technical duplicates, and the average values were used for subsequent statistical analysis.

### 2.4 Statistical analysis

For proteomic analysis of clinical bone marrow (BM) samples, proteins with missing values (peak intensity = 0) in >50% of samples were excluded. The remaining missing values were imputed using the sequential k-nearest neighbors (KNN) algorithm. All experiments were performed with three biological replicates.

Differentially expressed proteins (DEPs) were identified using a two-tailed unpaired Student’s *t*-test, followed by volcano plot analysis (|log*2* fold change| > X, *P* < 0.05) implemented in R (v3.3.2). Statistical significance was further validated via two-sided unpaired *t*-tests in GraphPad Prism 6.0. Following false discovery rate (FDR) adjustment using the Benjamini-Hochberg method to account for proteomic comparisons, updated significance threshold to FDR-adjusted *p*-value < 0.05.

Data are presented as mean ± standard deviation (SD) from three independent experiments. The sample size (*n*) represents biological replicates, and a *P-*value < 0.05 was considered statistically significant.

## 3 Results

### 3.1 Clinical characteristics of study cohorts

The demographic and clinical features of the study populations are presented in Supplementary Table 1 (bone marrow cohort) and Supplementary Table 2 (peripheral serum cohort). The bone marrow analysis included 33 newly diagnosed MM (NDMM) patients and 16 relapsed/refractory MM (RRMM) cases. The peripheral serum evaluation comprised four distinct groups: 36 NDMM patients, 36 MM patients in remission, 13 RRMM patients, and 36 healthy controls.

Key clinical parameters including immunoglobulin heavy/light chain isotype distribution showed no statistically significant differences between comparative groups (*p* > 0.05 by χ^2^ test). All enrolled patients underwent comprehensive clinical staging using both the Durie-Salmon (DS) and International Staging System (ISS) criteria to ensure standardized disease classification.

### 3.2 Workflow for the plasma proteome analysis

**FIGURE 1:**
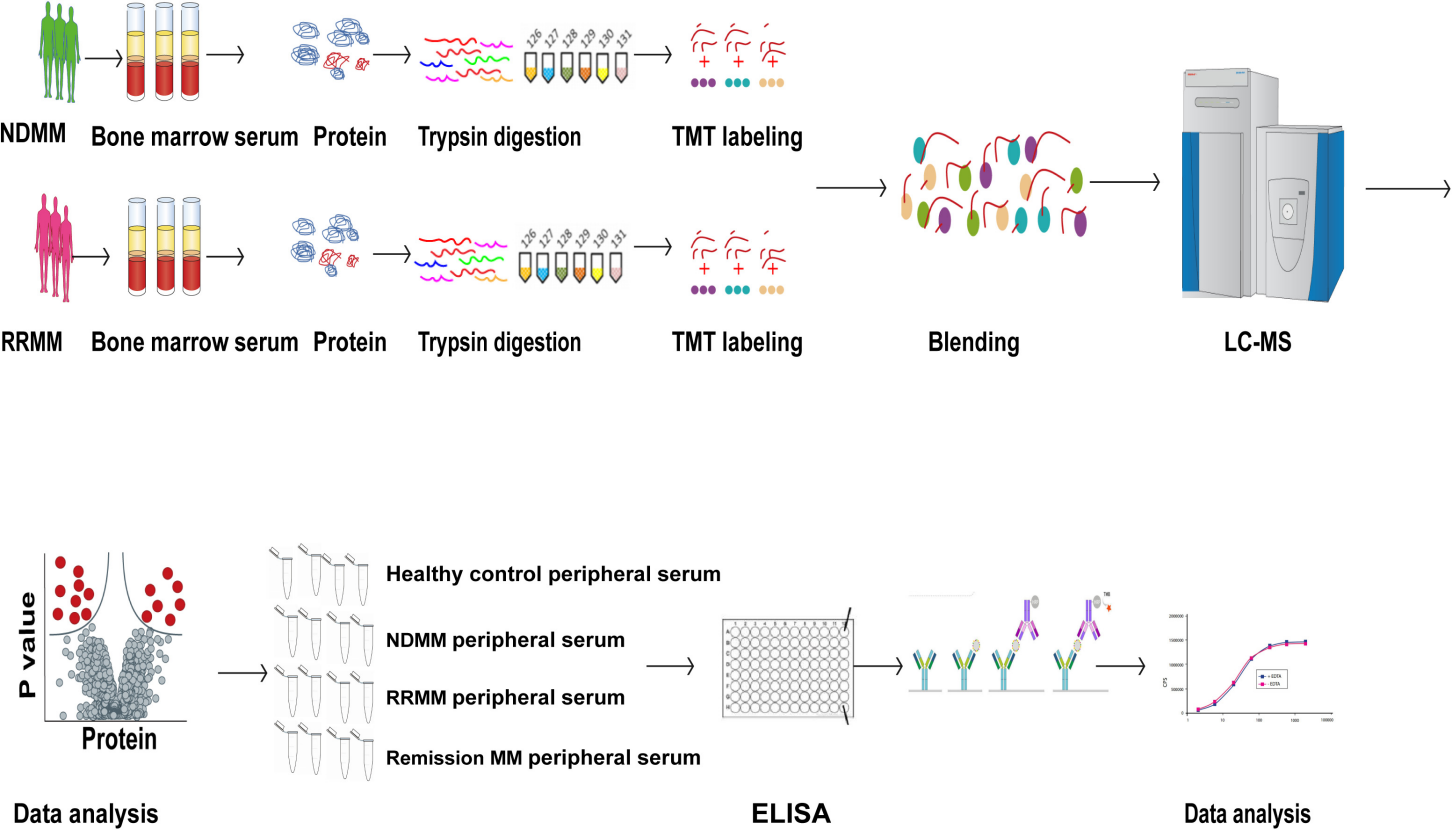
Schematic of the experimental design for quantitative proteomic analysis of newly diagnosed (NDMM) and relapsed/refractory multiple myeloma (RRMM).

Figure 1 illustrates the comprehensive workflow employed for comparative plasma proteome profiling between newly diagnosed multiple myeloma (NDMM) and relapsed/refractory multiple myeloma (RRMM) patients. Our quantitative proteomic analysis encompassed serum samples from nine NDMM cases and eight RRMM patients, selected based on stringent clinical criteria.

### 3.3 Proteomic profiling reveals distinct molecular signatures in RRMM

#### 3.3.1 Comprehensive protein identification and quantification

**FIGURE 2:**
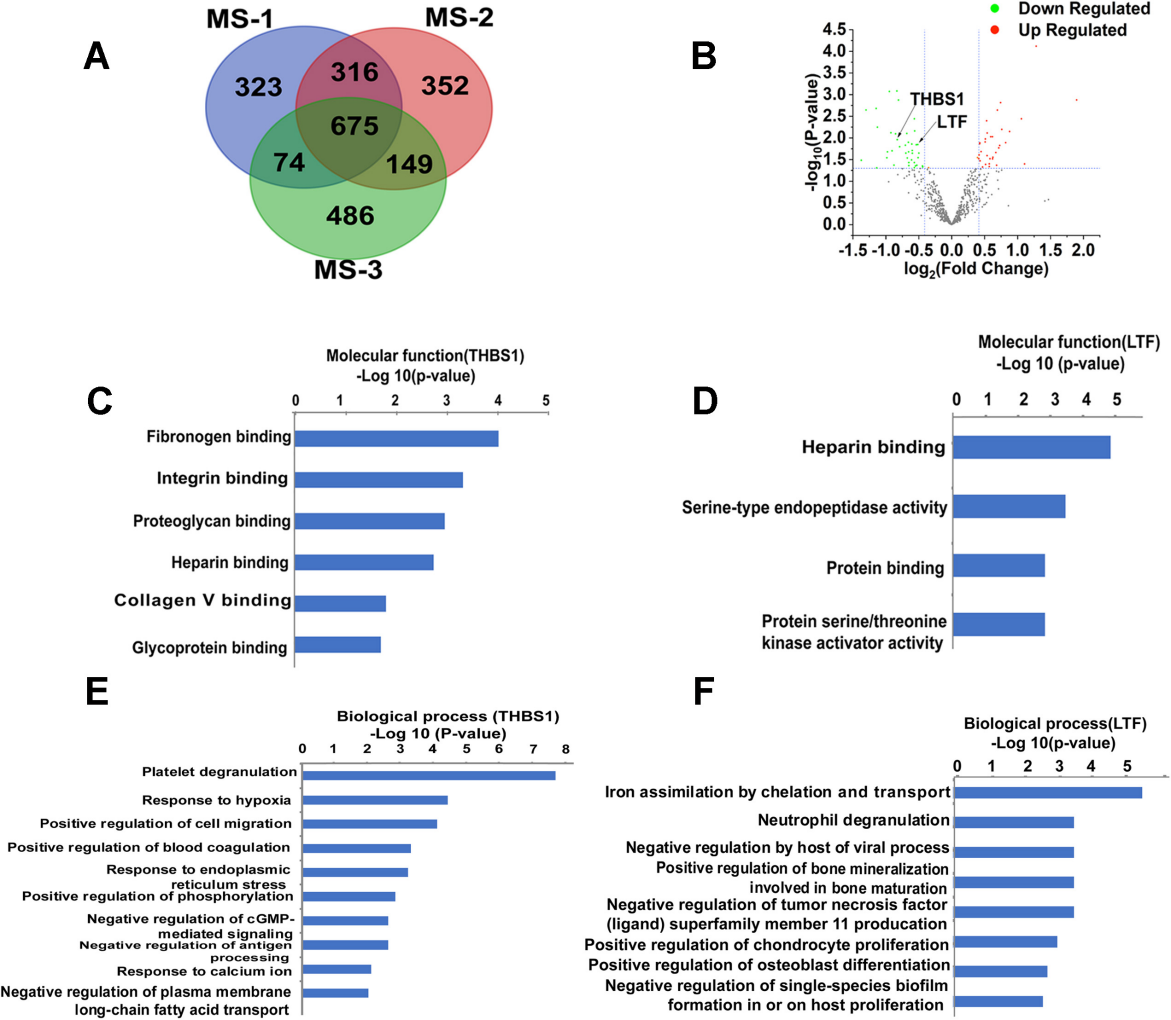
Mass spectrometry-based analysis of bioinformatics data. **(A)** The expression 97 proteins were significantly changed in the Venn diagram. **(B)** Thirty-four proteins were significantly upregulated in the volcano map, whereas 41 proteins were significantly downregulated. **(C)** The molecular function of THBS1. **(D)** The molecular function of LTF. **(E)** The major biological processes of THBS1. **(F)** The major biological processes of THBS1. –Log P, negative logarithm of the *P* value. The y-axis shows GO categories, and the x-axis shows –Log P. A larger –Log P indicates a smaller *p*-value. MS-1: The first mass spectrometry-based analysis;MS-2:The second mass spectrometry-based analysis; MS-3: The third mass spectrometry-based analysis.

Our large-scale proteomic analysis successfully identified an average of 1,380 high-confidence proteins across three independent mass spectrometry experiments (Figure 2A). Intersection analysis demonstrated remarkable consistency, with 675 proteins (48.9% of total identifications) being reproducibly detected in all analyzed samples (Figure 2A). This core proteome formed the basis for our subsequent differential expression analysis.

#### 3.3.2 Differentially expressed proteins in RRMM

Comparative quantification between RRMM and NDMM groups revealed significant alterations in protein abundance (Figure 2B). Specifically, we identified: 34 significantly upregulated proteins (≥1.3-fold change, *p* < 0.05), 41 significantly downregulated proteins (≤0.75-fold change, *p* < 0.05).

#### 3.3.3 Functional characterization of key biomarkers

Following false discovery rate (FDR) adjustment using the Benjamini-Hochberg method to account for multiple comparisons, both candidate biomarkers demonstrated statistically significant differential expression: THBS1: FDR-adjusted *p*-value (*q*-value) = 0.043, LTF: FDR-adjusted *p*-value (*q*-value) = 0.032.

Molecular function analysis demonstrated THBS1’s involvement in the fibrinogen binding, integrin binding (Figure 2C). Biological pathway analysis revealed THBS1’s participation in: Platelet degranulation (via interaction with TLN1, VCL, PLEK, MYH9, ILK, and ITGA2B). Cellular response to hypoxia (through PKLR, PDLIM1, F7, and PKM). Positive regulation of cell migration (mediated by F7, MMP9, ILK, and FERMT3) (Figure 2E).

GO analysis identified LTF’s molecular functions as: Heparin binding, Serine-type endopeptidase activity (Figure 2D). Key biological processes involving LTF included: Neutrophil degranulation (through PPBP, CECR1, MMP9, PGM1, and PKM), Negative regulation of TNFSF11 production (Figure 2F).

### 3.4 Validation of THBS1 and LTF as potential biomarkers for RRMM

#### 3.4.1 Biochemical validation of proteomic findings

To confirm our mass spectrometry results, we performed quantitative ELISA analysis of THBS1 and LTF expression patterns in both bone marrow and peripheral blood samples. Our integrated approach demonstrated consistent downregulation of these biomarkers in RRMM patients across multiple analytical platforms.

#### 3.4.2 Bone marrow serum analysis

**FIGURE 3:**
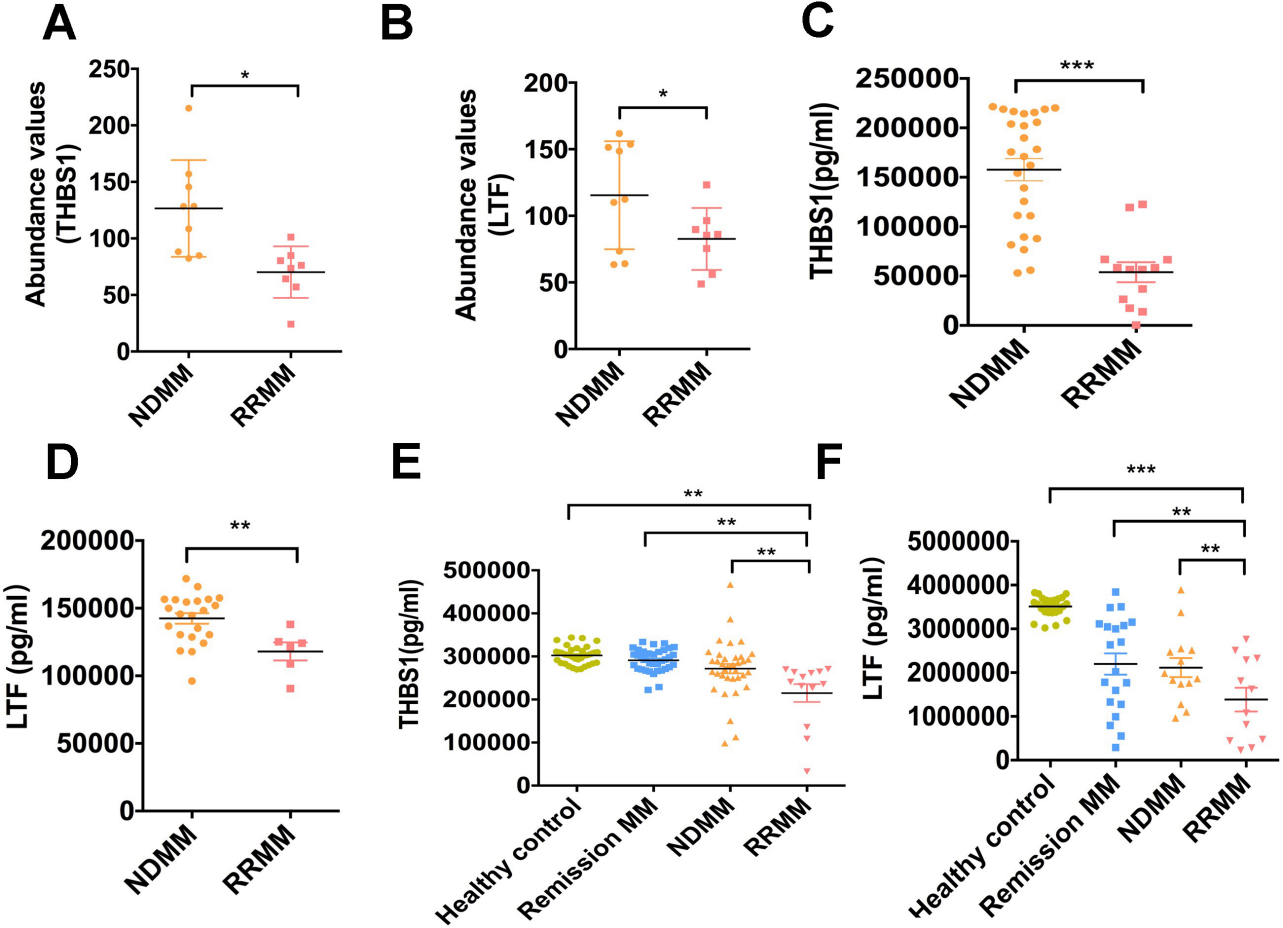
The expression of THBS1 and LTF in various stages of MM. **(A,B)** Mass spectrometry quantification of THBS1 and LTF in bone marrow serum from relapsed/refractory multiple myeloma (RRMM) (*n* = 8) and newly diagnosed (NDMM) (*n* = 9). THBS1: 70.19 ± 8.07 vs. 126.5 ± 14.26, *p* = 0.0047, LTF: 82.64 ± 8.24 vs. 115.40 ± 13.51, *p* = 0.048. **(C,D)** ELISA quantification of THBS1 and LTF in bone marrow serum. THBS1 (53,866 ± 10,194 pg/mL, *n* = 12; *p* < 0.0001) and LTF (117,998 ± 6,680 pg/mL, *n* = 6; *p* = 0.0064) in RRMM patients versus NDMM (THBS1: 157,690 ± 11,283 pg/mL, *n* = 24; LTF: 142,432 ± 3,885 pg/mL, *n* = 22). **(E,F)** Peripheral blood ELISA analysis of THBS1 and LTF levels at various stages of MM, including in healthy controls. THBS1 levels in healthy controls (302,398 ± 3,330 pg/mL, *n* = 36, reference group, NDMM: 271,730 ± 1,103 pg/mL, *n* = 36, remission phase 290,922 ± 4,394 pg/mL, *n* = 36), and relapsed/refractory cases 214,973 ± 20,705 pg/mL, *n* = 13). LTF concentrations in healthy controls (3,509,625 ± 45,663 pg/mL, *n* = 25, NDMM 2,114,306 ± 219,170 pg/mL, *n* = 14, remission 2,197,247 ± 243,406 pg/mL, *n* = 20 and relapse 1,384,292 ± 272,390 pg/mL, *n* = 12). All pairwise comparisons were statistically significant (*p* < 0.05). Data represent mean ± SD of *n* = biological replicates (*n* values as specified in Methods). Statistical analysis: Two-tailed unpaired *t*-test; *p < 0.05, **p < 0.01, ***p < 0.001 (exact *p*-values provided in Supplementary Table 3, 4).

Mass spectrometry quantification revealed significant reductions in both biomarkers: THBS1: 70.19 ± 8.07 (RRMM, *n* = 8) vs. 126.5 ± 14.26 (NDMM, *n* = 9); *p* = 0.0047, LTF: 82.64 ± 8.24 (RRMM) vs. 115.40 ± 13.51 (NDMM); *p* = 0.048 (Figures 3A, B).

ELISA validation in an expanded cohort confirmed these findings with greater sensitivity: THBS1: 53,866 ± 10,194 pg/mL (RRMM, *n* = 12) vs. 157,690 ± 11,283 pg/mL (NDMM, *n* = 24) *p* < 0.0001, LTF: 117,998 ± 6,680 pg/mL (RRMM, *n* = 6) vs. 142,432 ± 3,885 pg/mL (NDMM, *n* = 22) *p* = 0.0064 (Figures 3C, D and Supplementary Table 3).

### 3.4.3Peripheral blood profiling reveals stage-dependent alterations

Comprehensive ELISA analysis of peripheral blood samples demonstrated progressive decreases in biomarker levels corresponding to disease progression:

THBS1 Concentration Dynamics:

- Healthy controls: 302,398 ± 3,330 pg/mL (*n* = 36)
- NDMM: 271,730 ± 1,103 pg/mL (*n* = 36)
- Remission: 290,922 ± 4,394 pg/mL (*n* = 36)
- RRMM: 214,973 ± 20,705 pg/mL (*n* = 13)
- (all intergroup comparisons *p* < 0.05)

LTF Concentration Patterns:

- Healthy: 3,509,625 ± 45,663 pg/mL (*n* = 25)
- NDMM: 2,114,306 ± 219,170 pg/mL (*n* = 14)
- Remission: 2,197,247 ± 243,406 pg/mL (*n* = 20)

RRMM: 1,384,292 ± 272,390 pg/mL (*n* = 12)

(all intergroup comparisons *p* < 0.05) (Figures 3E, F and Supplementary Table 4).

## 4 Discussion

Relapsed/refractory multiple myeloma (RRMM) frequently presents significant diagnostic challenges during its initial phases, often manifesting either asymptotically or with non-specific clinical symptoms. This diagnostic ambiguity frequently leads to delayed clinical intervention, contributing substantially to myeloma-associated mortality rates. The limitations of current gold-standard diagnostic approaches are becoming increasingly apparent, for example, bone marrow biopsies, while diagnostically valuable, represent invasive procedures with inherent risks, or single-site sampling may not accurately reflect disease heterogeneity, and the impracticality of repeated invasive testing limits dynamic disease monitoring. Circulating biomarkers offer a paradigm shift in RRMM management through comprehensive disease profiling, systemic representation of tumor burden across all metastatic sites. Enables minimally invasive, repeatable sampling, facilitates pre-clinical detection of molecular relapse, allows for real-time therapeutic monitoring.

The past decade has witnessed remarkable progress in high-throughput analytical technologies: next-generation proteomic platforms with enhanced sensitivity and specificity, advanced mass spectrometry techniques enabling comprehensive protein profiling, sophisticated bioinformatics tools for complex data analysis, integrated multi-omics approaches combining genomic and proteomic data. These technological advancements have transformed biomarker discovery, allowing for: simultaneous screening of hundreds of potential biomarkers, identification of complex biomarker signatures, development of clinically actionable diagnostic panels, making composite screening of biomarkers possible (8). However, it has rarely been reported that serum biomarkers are used to assess disease progression in MM.

Through comprehensive quantitative proteomic analysis of serum samples from newly diagnosed multiple myeloma (NDMM) and relapsed/refractory multiple myeloma (RRMM) patients, we identified significant alterations in protein expression patterns associated with disease progression. Our investigation revealed: 34 significantly upregulated proteins (fold change ≥1.3, *p* < 0.05), 41 significantly downregulated proteins (fold change ≤0.75, *p* < 0.05) in bone marrow serum samples of RRMM patients compared to NDMM controls.

**FIGURE 4:**
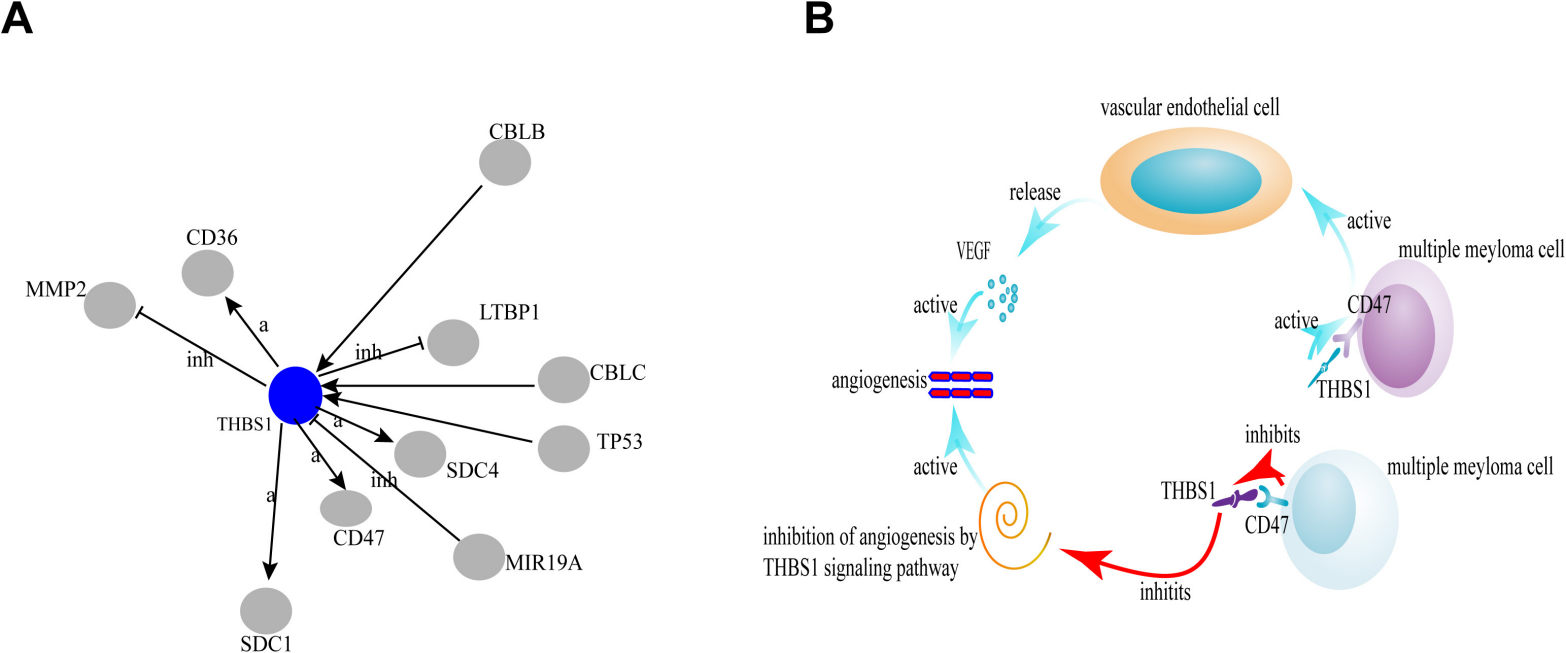
The correlation of THBS1 and CD47 by global signal transduction network. The blue circles represent the down-regulated genes. Interaction between the genes is shown as: a, activation; inh, inhibition; ex, expression.

In our study, by analyzing the quantitative proteome of serum from patients with NDMM and RRMM, we identified 34 upregulated and 41 downregulated proteins in the bone marrow samples. Two biomarkers, including THBS1 and LTF, showed lower expression level than that of NDMM, indicating that changes in the expression of two biomarkers may be related to the progression of MM. THBS1 is a multifunctional glycoprotein that serves as an inhibitor of VEGF and suppress of endothelial cell growth (9), and miR-21 targets THBS1 transcripts to promote endothelial regeneration (10). Patients with MM and higher THBS1 levels in bone marrow serum are more likely to achieve complete or very good partial responses after chemotherapy (11). The correlation of THBS1 deficiency with angiogenesis has long been proposed, but a clear mechanism has not been elucidated. THBS1 can affect tumor cell function through interactions with cell surface receptors. GO analysis of the proteomics data revealed that THBS1 at low levels activates CD47 (Figure 4A), which promoted angiogenesis (12). CD47 is associated with VEGFR, which is another proximal lateral binding partner, in endothelial cells (3); however, THBS1 induces disassociation of this complex and inhibits VEGFR2 signaling through CD47 to indirectly regulate tumor growth (13), beside we infer that CD47 inhibits THBS1, while inactivated THBS1 can inhibit an important signaling pathway: inhibition of angiogenesis by THBS1 signaling pathway, thereby activating angiogenesis (Figure 4B), and we will further confirm in the future. In addition, THBS1/CD47 signaling also control tumor perfusion by indirectly regulating tumor blood flow, thereby limiting tumor growth. Meanwhile, THBS1/CD47 interactions also activate NF-κB–RANK ligand signaling to increase malignancy and metastasis in myeloma cells (14).

Up to date, LTF dysregulation is described in many tumors, except for MM samples. LTF is an iron binding transport protein and its engineered overexpression in soft tissue sarcomas was shown to be associated with metastasis (15). However, in a prior study, LTF expression exhibited a negative correlation with oral tumor metastasis (16), which maybe by inhibiting TLR9 signaling (17). Furthermore, a recent analysis identified silencing LTF mediated breast cancer cell lines susceptibility (18, 19). In the present cohort, stable expression or overexpression of lactoferrin results in significant inhibition of human nasopharyngeal carcinoma and breast cancer cell growth, and reduces the possibility of tumor formation *in vivo* after orthotopic transplantation (20, 21). The exact mechanisms of LTF anticancer effect is unknown, however LTF mainly involved in the extracellular effects, intracellular effects, and immune stimulation (22), which may be a key factor in the mechanisms of action involved in cancer prevention.

## 5 Conclusion

Our systematic proteomic approach has identified THBS1 and LTF as promising biomarker candidates for MM progression monitoring. Their consistent downregulation in RRMM patients, coupled with their known biological functions, suggests potential roles in disease pathogenesis while offering clinically accessible markers for disease monitoring. These findings warrant further investigation into the mechanistic roles of THBS1 and LTF in MM progression, their utility in clinical decision-making algorithms, potential therapeutic applications targeting these pathways, and providing a foundation for developing minimally invasive, protein-based monitoring strategies to improve clinical management of MM patients.

## Data Availability

The datasets presented in this study can be found in online repositories. The names of the repository/repositories and accession number(s) can be found in the article/Supplementary material.

## Footnotes

1
http://proteomecentral.proteomexchange.org
