# Supplementary material for: Serum proteome profiling identified thrombospondin-1 and lactoferrin as biomarkers of relapsed multiple myeloma
Source: Front Med (Lausanne). 2025 Sep 8;12:1640245. doi: 10.3389/fmed.2025.1640245 (PMC12450997; doi:10.3389/fmed.2025.1640245)
Supplement: Supplementary file 1 [file Table_1.docx]

Supplemental Table S1

Clinical characteristic of NDMM and RRMM patients with BM serum

| Characteristic | NDMM  (n=33) | RRMM  （n=16） | NDMM vs RRMM  P |
| --- | --- | --- | --- |
| Age, n, % | 65(44-80) | 59(53-76) | NS |
| Sex：male/female | 15/18 | 4/12 | NS |
| Clinical features |  |  |  |
| Heavy chain, n, % |  |  | NS |
| No expression | -- | -- |  |
| Ig G | 22(66.6%) | 14(87.5%) |  |
| Ig A | 7(21.2%) | -- |  |
| Ig M | 1(3%) | -- |  |
| Ig D | 2(6%) | 2(12.5%) |  |
| Ig E | 1(3%) | -- |  |
| Light chain, n, % |  |  |  |
| k | 17(51.5%) | 8(50%) |  |
| λ | 16(48.4 %) | 8(50%) |  |
| Durie-Salmon stage n, % |  |  |  |
| I | 3(9%) | 1(6.2%) |  |
| II | 4(12.1%) | -- |  |
| III | 26(78.8 %) | 15(93.75%) |  |
| ISS stage, n, % |  |  |  |
| I | 2(6%) | 3(18.7%) |  |
| II | 5(15%) | 5(31.2%) |  |
| III | 26(78.8%) | 8(50%) |  |

Abbreviations: NDMM: newly diagnosed multiple myeloma；RRMM：relapsed multiple myeloma; BM: bone marrow; NS, not significant
