# Supplementary material for: Serum proteome profiling identified thrombospondin-1 and lactoferrin as biomarkers of relapsed multiple myeloma
Source: Front Med (Lausanne). 2025 Sep 8;12:1640245. doi: 10.3389/fmed.2025.1640245 (PMC12450997; doi:10.3389/fmed.2025.1640245)
Supplement: Supplementary file 2 [file Table_2.docx]

Supplemental Table S2

Clinical characteristic of NDMM, remission MM, RRMM patients and healthy people with PB serum

| Characteristic | NDMM | Remission MM | RRMM | Normal people |
| --- | --- | --- | --- | --- |
| Age, n, % | 63(41-82) ^NS^ | 60.7(34-76) ^NS^ | 55.3(47-65) ^NS^ | 56.6(52-70) ^NS^ |
| Se：male/female | 14/22 ^NS^ | 20/16 ^NS^ | 5/8 ^NS^ | 11/25 ^NS^ |
| Clinical features | NS | NS | NS |  |
| Heavy chain, n, % |  |  |  |  |
| No expression | 4(11.1%) | 2(5%) | 2(15.4%) |  |
| Ig G | 19(52.7%) | 18(50%) | 9(69.2%) |  |
| Ig A | 8(22.2%) | 13(36%) | 2(15.4%) |  |
| Ig M | -- | -- | -- |  |
| Ig D | 5(13.8%) | 3(8%) | -- |  |
| Ig E | -- | -- | -- |  |
| Light chain, n, % |  |  |  |  |
| k | 15(41.6%) | 21(58.3%) | 5(38.4) |  |
| λ | 17(47.2%) | 13(36.1%) | 6(46.2%) |  |
| No expression | 4(11%) | 2(5%) | 2(15.3) |  |
| Durie-Salmon stage n, % |  |  |  |  |
| I | 6(16%) | 3（8.3%） | -- |  |
| II | 2（5%） | 2（5.5%） | 2（15.3%） |  |
| III | 28（77%） | 31(86.1%） | 11（84.6%） |  |
| ISS stage, n, % |  |  |  |  |
| I | 11（30.5%） | 9（25%） | -- |  |
| II | 9（25%） | 6（16.6%） | 7（53.8%） |  |
| III | 16（16.5%） | 21（58.3%） | 6（46.2%） |  |

Abbreviations: NDMM: newly diagnosed multiple myeloma；RRMM：relapsed multiple myeloma; Remission MM：remission multiple myeloma; PB: peripheral blood; NS, not significant
