# Supplementary material for: Serum proteome profiling identified thrombospondin-1 and lactoferrin as biomarkers of relapsed multiple myeloma
Source: Front Med (Lausanne). 2025 Sep 8;12:1640245. doi: 10.3389/fmed.2025.1640245 (PMC12450997; doi:10.3389/fmed.2025.1640245)
Supplement: Supplementary file 3 [file Table_3.docx]

Supplemental Table S3

The expression of biomarker in the bone marrow

|  | NDMM | RRMM | NDMM vs RRMM  P |
| --- | --- | --- | --- |
| ¶ THBS1 | 126.5±14.26(n=9) | 70.19±8.07(n=8) | P=0.0047 |
| ¶ LTF | 115.4±13.51(n=9) | 82.64±8.24(n=8) | P=0.048 |
| § THBS1 | 157690±11283（n=24） | 53866±10194(n=12) | P＜0.0001 |
| § LTF | 142432±3885(n=22) | 117998±6680(n=6) | P=0.0064 |

Abbreviations: RRMM: relapsed multiple myeloma; NDMM: newly diagnosed multiple myeloma; THBS1: Thrombospondin-1; LTF: Lactoferrin. ¶ Mean abundance values of biomarker by mass spectrum. § The concentration of biomarker in the bone marrow

Supplementary Table S3. Thrombospondin-1 (THBS1) and lactoferrin (LTF) concentrations in bone marrow serum samples as measured via the enzyme-linked immunosorbent assay.
