# Supplementary material for: Serum proteome profiling identified thrombospondin-1 and lactoferrin as biomarkers of relapsed multiple myeloma
Source: Front Med (Lausanne). 2025 Sep 8;12:1640245. doi: 10.3389/fmed.2025.1640245 (PMC12450997; doi:10.3389/fmed.2025.1640245)
Supplement: Supplementary file 4 [file Table_4.docx]

Supplemental Table S4

The concentration of biomarker in the PB serum (p g/ml)

|  | THBS1 | LTF |
| --- | --- | --- |
| Healthy control | 302398±3330^***^（n=36） | 3509625±45663^***^ (n=25) |
| NDMM | 271730±1103^*^（n=36） | 2114306±219170^*^ (n=14) |
| Remission MM | 290922±4394^**^（n=36） | 2197247±243406^*^ (n=20) |
| RRMM | 214973±20705（n=13） | 1384292±272390 (n=12) |

Abbreviations: Remission MM: remission multiple myeloma; PB: peripheral blood

*P value less than 0.05; **p value less than 0.001; ^***^ p value less than 0.0001

Supplementary Table S4. Thrombospondin-1 (THBS1) and lactoferrin (LTF) concentrations in the peripheral blood samples as determined via the enzyme-linked immunosorbent assay.
